# A DNA vaccine against tuberculosis based on the 65 kDa heat-shock protein differentially activates human macrophages and dendritic cells

**DOI:** 10.1186/1479-0556-6-3

**Published:** 2008-01-21

**Authors:** Luís H Franco, Pryscilla F Wowk, Célio L Silva, Ana PF Trombone, Arlete AM Coelho-Castelo, Constance Oliver, Maria C Jamur, Edson L Moretto, Vânia LD Bonato

**Affiliations:** 1 Núcleo de Pesquisas em Tuberculose, Departamento de Bioquímica e Imunologia, Faculdade de Medicina de Ribeirão Preto da Universidade de São Paulo. Av. Bandeirantes, 3900, 14049-900, Ribeirão Preto, SP, Brasil; 2 Laboratório de Fracionamento e Estoque – Centro Regional de Hemoterapia do Hospital das Clínicas, Faculdade de Medicina de Ribeirão Preto da Universidade de São Paulo. Rua Tenente Catão Roxo 2501, Ribeirão Preto, SP, Brasil; 3 Departamento de Biologia Celular e Molecular e Bioagentes Patogênicos, Faculdade de Medicina de Ribeirão Preto da Universidade de São Paulo. Av. Bandeirantes, 3900, 14049-900, Ribeirão Preto, SP, Brasil

## Abstract

**Background:**

A number of reports have demonstrated that rodents immunized with DNA vaccines can produce antibodies and cellular immune responses presenting a long-lasting protective immunity. These findings have attracted considerable interest in the field of DNA vaccination. We have previously described the prophylactic and therapeutic effects of a DNA vaccine encoding the *Mycobacterium leprae *65 kDa heat shock protein (DNA-HSP65) in a murine model of tuberculosis. As DNA vaccines are often less effective in humans, we aimed to find out how the DNA-HSP65 stimulates human immune responses.

**Methods:**

To address this question, we analysed the activation of both human macrophages and dendritic cells (DCs) cultured with DNA-HSP65. Then, these cells stimulated with the DNA vaccine were evaluated regarding the expression of surface markers, cytokine production and microbicidal activity.

**Results:**

It was observed that DCs and macrophages presented different ability to uptake DNA vaccine. Under DNA stimulation, macrophages, characterized as CD11b^+^/CD86^+^/HLA-DR^+^, produced high levels of TNF-alpha, IL-6 (pro-inflammatory cytokines), and IL-10 (anti-inflammatory cytokine). Besides, they also presented a microbicidal activity higher than that observed in DCs after infection with *M. tuberculosis*. On the other hand, DCs, characterized as CD11c^+^/CD86^+^/CD123^-^/BDCA-4^+^/IFN-alpha^-^, produced high levels of IL-12 and low levels of TNF-alpha, IL-6 and IL-10. Finally, the DNA-HSP65 vaccine was able to induce proliferation of peripheral blood lymphocytes.

**Conclusion:**

Our data suggest that the immune response is differently activated by the DNA-HSP65 vaccine in humans. These findings provide important clues to the design of new strategies for using DNA vaccines in human immunotherapy.

## Background

DNA vaccination has arisen as a safe and effective strategy for inducing protective cell and humoral immunity in preclinical models of infectious diseases [1,2]. These vaccines are able to activate the innate immune system, even in the absence of an adjuvant. It is assumed that they interact with the pattern recognition receptor Toll-like receptor 9 (TLR9) through unmethylated CpG oligodeoxynucleotides (CpG ODNs) present on plasmid backbone [3,4]. Downstream, TLR9 interacts with the adaptor molecule MyD88 (myeloid differentiation factor 88), and the activation of MyD88 leads to the activation of several transcription factors, resulting in the up-regulation of cytokine and chemokine gene expression [5-8]. In relation to adaptive immune response, there are at least three mechanisms by which the antigen encoded by plasmid DNA is processed and presented to elicit immune response: (I) direct priming by somatic cells [9]; (II) direct transfection of professional antigen-presenting cells (APCs) [10-12]; and (III) cross-priming in which plasmid DNA transfects a somatic cell and/or a professional APC and the secreted protein is taken up by other professional APC and presented to T cells [13-16].

Early studies conducted in mice showed that DNA vaccination conferred protection against pathogen challenge [17-20]. Experimental data collected by our group over the last few years have shown that the DNA vaccine encoding the *Mycobacterium leprae *65 kDa heat shock protein (DNA-HSP65) has prophylactic and therapeutic effects in a murine model of TB [17,19,21,22]. The prophylactic effect initially obtained from this vaccine was equal to that elicited by live BCG vaccine in mice and this protection was associated with the presence of CD8^+^/CD44^hi ^IFN-gamma – producing cytotoxic cells [17,19].

Additionally, we demonstrated that DNA vaccine can be taken up by CD11b^+^(macrophages) and CD11c^+ ^(DC) cells, as well as by B lymphocytes after its administration in mice [23]. However, several studies in nonhuman primates and human clinical trials have suggested that DNA vaccines are not nearly as immunogenic in these species as they are in rodents [24-27]. Therefore, a better understanding of how DNA-HSP65 vaccine activates human immune response was taken into account herein.

Thus, the aim of this study was to compare the immune responses of human macrophages and DCs induced byDNA-HSP65 vaccine. These professional APCs drive the activation of T lymphocytes and are thought to be the most important stimulators of adaptive immune response to antigens. We compared the immune response induced by DNA-HSP65 vaccine *in vitro *through the evaluation of surface markers, cytokine production and microbicidal activity of human macrophages and DCs. Additionally, the capacity of DNA-HSP65 to activate the adaptive immune response was evaluated. The data reported herein provide important implications for the design of new vaccination strategies, which may contribute to the use of DNA plasmid in human immunotherapy.

## Methods

### Monoclonal antibodies

The mAbs specific for CD80 (clone BB1) coupled to fluorescein isothiocyanate (FITC), CD86 (clone IT2.2), HLA-DR (clone G46-6), CD83 (clone HB15e), coupled to phycoerythrin (PE), CD11b (clone ICRF44), CD11c (clone B-ly6), and CD123 (clone 9F5) coupled to Cy-chrome, were purchased from BD (BD, San Diego, CA, USA). The mAbs specific for CD1c (clone AD5-8E7) and BDCA-4 (clone AD5-17F6) coupled to PE were obtained from Miltenyi Biotec (Auburn, CA, USA). The purified mAb TLR9 (26C593 clone) was obtained from Imgenex (San Diego, CA, USA), and the biotinylated anti-mouse IgG was obtained from Bioscience (Toronto, Canada).

### Plasmid construction and purification

DNA-HSP65 vaccine was derived from pVAX vector (Invitrogen, Carlsbad, CA, USA), which had previously been digested with *BamHI *and *Not I *(Invitrogen), and a 3.3-kb fragment (corresponding to the *M. leprae *HSP65 gene) was inserted. The vector pVAX was used as a control. Plasmids were replicated in DH5alpha *Escherichia coli *and purified with Endofree Plasmid Giga kit (Qiagen, Valencia, CA, USA) according to the manufacturer's protocol. Endotoxin levels were determined using a QCL-1000 Limulus amoebocyte lysate kit (Cambrex Company, Walkersville, MD, USA), and were less than 0.1 endotoxin units (EU)/μg DNA.

### Plasmid DNA labelling

The DNA vaccine was labeled with Alexa Fluor 594 or Alexa Fluor 488 by Universal Linkage System (ULS™) using the ULYSIS nucleic acid labelling kit (Invitrogen, Molecular Probe) as previously described [23]. The conformation of labeled plasmid was not altered.

### Cell cultures

Peripheral blood mononuclear cells (PBMCs) were obtained from blood donated by healthy volunteers at the *Fundação Hemocentro de Ribeirão Preto *(Ribeirão Preto Haemocentre Foundation, Ribeirão Preto, Brazil). This work was approved by Comitê de Ética em Pesquisa do Hospital das Clínicas de Ribeirão Preto (Ethic Committee Research from Ribeirão Preto Clinical Hospital, Brazil). Mononuclear cells were separated by density gradient centrifugation using Ficoll-Paque (GE Life Sciences, Uppsala, Sweden). Monocytes were purified by density gradient centrifugation using Percoll (GE Life Sciences). Macrophages and DCs were differentiated by culturing monocytes in 24-well tissue culture plates (Corning, Corning, NY, USA) with 1 ng/mL of GM-CSF (BD) or with 14 ng/mL of IL-4 (BD) and 7 ng/mL of GM-CSF, respectively, for 7 days at approximately 1 × 10^6 ^cells/mL in RPMI 1640 (Sigma-Aldrich) supplemented with 10% foetal bovine serum (FBS) (Invitrogen, Gibco), streptomycin/ampicillin (Invitrogen, Gibco) and gentamicin (Invitrogen, Gibco). Plasmacytoid dendritic cells (pDCs) were purified from PBMCs by positive selection with immunomagnetic microbeads (Miltenyi Biotec, Auburn, CA, USA) based on BDCA-4 expression.

### Fluorescence microscopy

An amount of 5 × 10^4 ^macrophages and DCs were stimulated with 5 μg of Alexa Fluor 594-labeled DNA vaccine for 4 h for uptake assays. These cells were mounted on glass coverslips with Cell-tak (BD, New Bedford, MA, USA) with Fluormount-G (Electron Microscopy Sciences) and analysed with a Nikon Eclipse E800 fluorescence microscope (Nikon USA, Melville, NY). Images were acquired with a Nikon DXM-1200 digital camera (Nikon USA) connected to the microscope.

### Confocal microscopy

Macrophages, myeloid and plasmacytoid DCs were placed onto Cell-Tak-coated glass coverslips (BD Biosciences, New Bedford, MA, USA), fixed with 4% paraformaldehyde (Electron Microscopy Sciences, Fort Washington, PA, USA) for 15 min at 37°C, and permeabilised with 0.3% Triton X-100 (Sigma-Aldrich) for 10 min at 25°C. The cells were washed with 0.1 M glycine (Sigma-Aldrich) for 5 min, and then labeled with purified mAb anti-TLR9 (5 μg/mL) (Imgenex) for 30 min at 4°C. Subsequently, the cells were incubated with biotinylated anti-mouse IgG (7.5 μg/mL; Bioscience) for 1 h at room temperature. Finally, cells were incubated with streptavidin conjugated to Alexa Fluor 488 (Molecular Probes, Eugene, OR, USA) for 30 min, mounted on glass slides with Fluormount (Electron Microscopy Sciences) and examined with a Leica TCS SP2 AOBS (Leitz, Manheim, Germany).

### FACS analysis

Macrophages and DCs were stimulated with 20 μg/mL of DNA vaccine or DNA vector over a 48 h period to evaluate cell surface phenotype. Additionally, 500 ng/mL of LPS (*Salmonella typhimurium*, Sigma) was used as positive control of cellular activation. To study the capacity of the cells to uptake DNA, macrophages and DCs were stimulated with Alexa Fluor 488-labeled DNA vaccine for 1 h. Then, the cells were analysed by flow cytometry (FACSort, Becton Dickinson, San Jose, CA, USA). A biparametric gate in the forward (FSC) and side scatter (SSC) dot plot was drawn around the macrophages or DCs populations. Approximately 4000 Mac-1^+ ^(macrophages) or CD11c^+ ^(DC) cells were acquired. The computer analysis was made using the Cell-Quest program (version 3.3).

### Cytokine secretion

Supernatants from macrophages and DCs cultures stimulated with DNA vaccine, DNA vector or LPS were harvested at 48 h after stimulation. Cytokine levels were determined by ELISA using recombinant cytokines for generating standard curves. Purified mAb anti-TNF-alpha (clone Mab1), anti-IL-6 (clone MQ2-13A5), anti-IL-10 (clone JES3-19F1), anti-IL-12p40 (clone C8.3), as well as biotinylated mAb anti-TNF-alpha (clone Mab11), anti-IL-6 (clone MQ2-39C3), anti-IL-10 (clone JES3-12G8), and anti-IL-12p40 (clone C8.6) were obtained from BD and used according to the manufacturer's instructions. Additionally, supernatants from cultures of monocyte-derived DCs and peripheral blood pDCs were assayed for IFN-alpha detection by Interferon-alpha ELISA Kit, (Immuno-Biological Laboratories, Minneapolis, MN, USA).

### Culture of *M. tuberculosis *and infection of macrophages and DCs

*M. tuberculosis *H37Rv (ATCC n° 27294) was obtained from an aliquot frozen at -70°C. Fifty microliters of this aliquot (viability greater than 85%) were cultured in Lowenstein-Jensen medium for 20–30 days at 37°C. *M. tuberculosis *was then added to 10 mL of 7H9 medium (Difco, BD, Detroit, USA) and incubated for 7–10 days at 37°C. After analysis of viability, the bacilli number was determined by optic density of the culture at 540 nm. The bacterial suspension was then centrifuged at 4000 × g for 20 min and the pellet was diluted in RPMI 1640 supplemented with 10% FBS and antibiotic-free. Macrophages and DCs were infected with *M. tuberculosis *with a multiplicity of infection (MOI) of 1 bacillus per 1 cell (MOI = 1). Four hours after infection the supernatants were removed, the cells were washed, centrifuged at 900 × g for 10 min and then lysed with a solution of 0,25% SDS-PBS (J.T. Baker, Phillipsburg, NJ, USA). Serial dilutions were plated in Middlebrook 7H11 agar medium. The same procedure was performed at 7 days after infection. The colony forming units (CFU) were counted after 20–30 days.

### RT-PCR for mRNA Hsp65 detection

Total RNA was isolated from PBMCs by extraction in Trizol Reagent (Invitrogen) and alcohol precipitation, followed by an additional treatment with DNAse I amplification grade (Invitrogen) to avoid genomic and plasmid DNA contamination. Total RNA (1 μg) was reverse transcribed using oligo(dT) primers and reverse transcriptase (Invitrogen) according to the manufacturer instructions. The PCR amplification was carried out using 3 μL of cDNA preparation and specific primer pairs of *M. leprae *Hsp65 (sense 5'-TCAAGGTGGCGTTGGAAGC-3' and antisense 5'-CCGTGACCCACTGAAAGGTTA-3'; giving a 103-bp band). Samples were submitted to 35 cycles of amplification in a PTC-200 Peltier Thermal Cycler (MJ Research Inc., Watertown, MA, USA). In each cycle, denaturation was performed at 95°C for 45 sec, primers were annealed to target cDNA at 65°C for 40 sec, and extension was carried out at 72°C for 90 sec. Messenger RNA for beta-actin was detected by PCR using cDNA and beta-actin-specific primers (sense 5' ATGTTTGAGACCTTCAACA-3' and antisense 5'-CACGTCAGACTTCATGATGG-3'; giving a 495-bp band). The PCR products were visualised by ultraviolet illumination after electrophoresis on a 1% agarose gel containing ethidium bromide.

### PBMC proliferation assay

A total of 2 × 10^5 ^PBMCs were stained with 5-(and-6)-carboxyfluorescein diacetate, succinimidyl ester (CFSE; Invitrogen, Molecular Probes) and plated in 96 well round bottom culture plate (Corning) in RPMI 1640 (Sigma-Aldrich) supplemented with 10% autologous serum, streptomycin/ampicillin (Invitrogen, Gibco) and gentamicin (Invitrogen, Gibco). PBMCs were cultured during 7 days with recombinant Hsp65 or during 12 days with DNA vaccine or vector. Additionally, PBMC were cultured with recombinant Hsp65 plus DNA vaccine or vector during 12 days. The cells were then harvested and evaluated for their CFSE content by flow cytometry. As positive control, PBMCs were stimulated with phytohemagglutinin. A gate in FSC and SSC dot plot was drawn around the lymphoblast population and the frequency of CFSE-containing cells was determined.

### Statistical analysis

Data are expressed as means ± SEM. Statistical significance of differences was determined by the unpaired Student's *t*-test. Differences which provided *P *< 0.05 were considered to be statistically significant. Statistical analyses were performed by using PRISM software (version 4.0; GraphPad, San Diego, CA, USA).

## Results

### Characterization of immature DCs and macrophages

**Figure 1:**
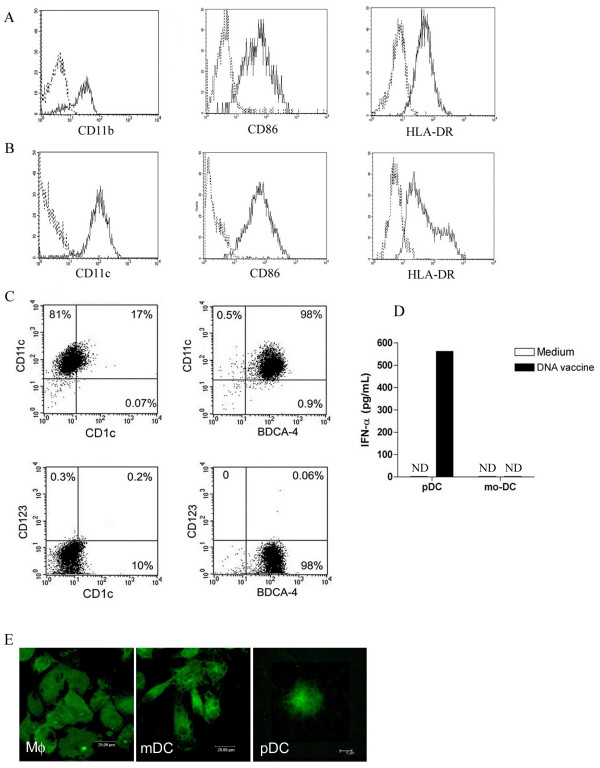
**Phenotypic characterization of monocyte-derived macrophages and DCs**. (A) Expression of markers CD11b (Mac-1), CD86 and HLA-DR on the surface of macrophages, and (B) CD11c, CD86 and HLA-DR on the surface of DCs was evaluated by flow cytometry (all markers are indicated by solid lines). Dotted-line histograms indicate isotype control mAb. These results are representative of seven independent experiments. (C) Expression of CD1c, CD123 (IL-3 receptor) and BDCA-4 on the surface of DCs. (D) IFN-alpha production by monocyte-derived DC (mo-DC) and plasmacytoid DC (pDC). These results are representative of three independent experiments. (E) Intracellular expression of TLR9 by macrophages and DCs analysed by confocal microscopy.

Freshly isolated monocytes cultured with GM-CSF differentiate into macrophages, whereas those cultured with GM-CSF plus IL-4 differentiate into DCs. As expected, macrophages and DCs differed morphologically. Macrophages were characterized as large and adherent cells, while DCs were round, smaller than macrophages and presented cytoplasmic extensions (dendrites) (data not shown). Macrophages and DCs were characterized as CD11b^+ ^and CD11c^+ ^cells, respectively. Both CD11b^+ ^and CD11c^+ ^cells constitutively expressed CD86 and HLA-DR molecules (Figures 1A and 1B). We further observed that 17% of DCs were characterized as CD11c^+^CD1c^+ ^and 98% were CD11c^+ ^BDCA-4^+^. CD123, a receptor exclusively expressed by plasmacytoid dendritic cells (pDCs), was not detected on the surface of either CD1c^+ ^or BDCA-4^+ ^cells (Figure 1C). Moreover, we also evaluated the IFN-alpha production by these cells. An experimental control was performed with pDCs. The pDCs stimulated with DNA vaccine secreted higher levels of IFN-alpha in comparison to the unstimulated pDCs (Figure 1D). On the other hand, monocyte-derived DCs did not secrete IFN-alpha.

In order to analyse the expression of TLR9 by macrophages and DCs, confocal microscopy was used (Figure 1E). It was found that monocyte-derived macrophages and DCs displayed strong cytoplasmic staining for TLR9, indicating its presence in intracellular compartments. pDCs isolated from peripheral blood were stained and used as positive control for TLR9 expression. These results were confirmed by flow cytometry analyses (data not shown).

### Uptake of DNA vaccine by macrophages and DCs

**Figure 2:**
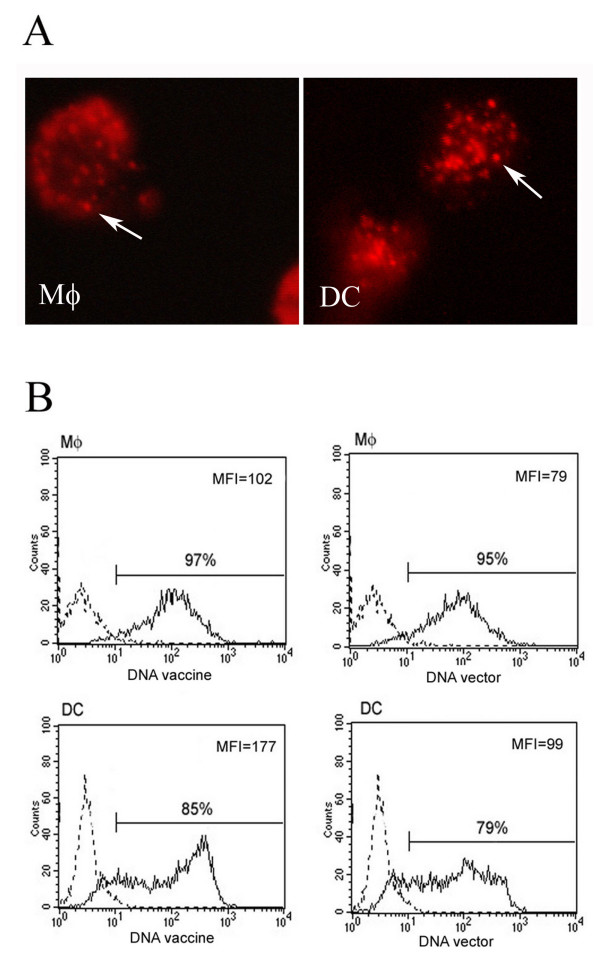
**Uptake of DNA-HSP65 by macrophages (Mφ) and DC**. (A) Cells were stimulated for 4 h with Alexa Fluor 594-labeled DNA-HSP65 and analysed by fluorescence microscopy. Endocytic vesicles are indicated by white arrows. (B) Differential capacity of macrophages and DCs to uptake DNA vaccine. Cells were stimulated for 1 h with Alexa Fluor 488-labeled DNA-HSP65 and analysed by flow cytometry. These results are representative of three independent experiments. Black line: stimulated cells; dotted line: non-stimulated cells.

To determine whether human macrophages and DCs would be able to taken up naked DNA-HSP65, we stimulated cells with fluorescent-labeled DNA vaccine. Four hours after stimulation with naked DNA-HSP65, fluorescent endocytic vesicles were observed in the cytoplasm of macrophages and DCs (Figure 2A), suggesting that the plasmid was taken up by these cells during this period. Flow cytometry analyses showed that macrophages and DCs had different ability to taken up naked DNA vaccine (Figure 2B). The DNA vaccine or vector was uptaken by almost 100% of macrophages CD11b^+ ^and by approximately 85% of DCs CD11c^+^. The analysis of median fluorescence intensity, which indicates the ability to take up DNA on a per-cell basis, show that DCs behaved with a bimodal pattern: while a subpopulation of CD11c+ cells displayed low uptake rates, the other one presented high uptake capacity. These values were similar when the cells were stimulated with either vaccine or vector. These results suggest that the uptake of DNA-HSP65 vaccine or vector by DCs was higher than that observed in macrophages.

### Activation of the innate immune response induced by DNA-HSP65

**Figure 3:**
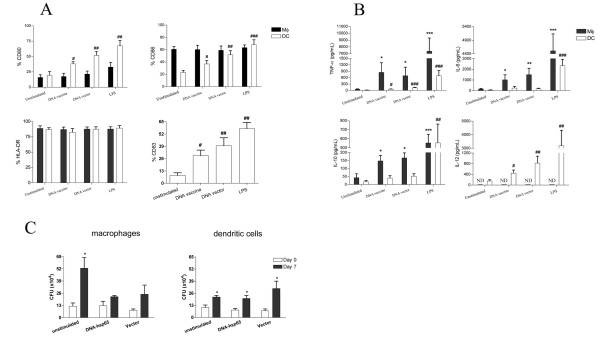
**Activation of the innate immune response mediated by DNA-HSP65**. (A) Expression of costimulatory molecules and HLA-DR on the surface of macrophages (Mφ) and DC stimulated with DNA vaccine. Cells were stimulated with DNA vaccine, DNA vector or LPS (positive control). After 48 h stimulation, the expression of surface molecules was evaluated by flow cytometry. Each column represents the mean percentage of Mφ or DC positive for CD80, CD86 or HLA-DR, or DC positive for CD83 ± SEM. Cells were obtained from 11 cultures of Mφ and 7–9 cultures of DC from different healthy individuals. (B) Mφ and DC were incubated for 48 h with DNA vaccine, DNA vector or LPS and the production of TNF-alpha, IL-6, IL-10 and IL-12p40 was evaluated. Each column represents the mean ± SEM of cytokine production detected in 6–8 Mφ cultures or 7–10 DC cultures obtained from healthy donors. *p < 0.05; **p < 0.01; ***p < 0.001, in relation to non-stimulated Mφ. #*p *< 0.05; ##*p *< 0.01; ###*p *< 0.001, in relation to non-stimulated DC. (C) Intracellular growth of *M. tuberculosis *in Mφ or DCs stimulated with DNA-HSP65. Mφ and DCs were stimulated with DNA vaccine or DNA vector (both at 20 μg/mL) for 48 h and infected with *M. tuberculosis *at MOI = 1. CFU numbers were determined at 4 h (day 0) and 7 days (day 7) after infection. Results represent the mean ± SEM of five experiments (for DCs) or three experiments (for Mφ). * p < 0,05, when compared to CFU numbers recovered on days 0 and 7 postinfection.

In order to study the activation of innate immune response mediated by DNA-HSP65, human macrophages and DCs stimulated with the DNA vaccine were evaluated regarding the cytokine production, expression of surface markers and microbicidal activity. In relation to cellular phenotype, we did not observe any variation in the number of DNA vaccine-stimulated macrophages expressing HLA-DR, CD80 or CD86 molecules (Figure 3A) or changes in the median fluorescence intensity (data not shown). Conversely, the stimulation of DCs with DNA vaccine resulted in an up-regulation of CD80, CD86 and CD83 (a maturation marker) expression (Figure 3A). After stimulation with DNA vaccine or DNA vector, no difference was seen in the number of DCs expressing HLA-DR. In all experiments LPS was used as positive control of cellular activation.

Regarding the cytokines production, macrophages stimulated with DNA vaccine secreted levels of TNF-alpha, IL-6 and IL-10 significantly higher than those of the unstimulated cells. Similar levels of these cytokines were secreted by vector-stimulated cells (Figure 3B). Notably, vaccine-stimulated macrophages did not produce either IL-12p40 (Figure 3B) or IL-12p70 (data not shown). In contrast, vaccine or vector-stimulated DCs provided significantly higher levels of TNF-alpha and IL-12p40 than those provided by the unstimulated cells. The production of TNF-alpha by DCs was also observed in experiments that were carried out with immunostimulatory CpG, an additional control (data not shown). DCs produced lower levels of TNF-alpha, IL-6 and IL-10 compared to macrophages.

To evaluate whether the activation induced by DNA-HSP65 could increase the microbicidal capacity of macrophages and DCs against *M. tuberculosis*, these cells were stimulated with DNA vaccine or vector and then were infected. Figure 3C shows that unstimulated macrophages were more permissive to *M. tuberculosis *growth when compared to macrophages that had been stimulated with DNA vaccine or DNA vector. On day 7 after infection, the bacterial load of the unstimulated infected macrophages differed significantly from that recovered on day 0 (after 4 h). However, the number of CFU recovered from macrophages that had previously been stimulated with DNA vaccine or vector was similar at 0 and 7 days postinfection. When we analysed the mycobacterial growth in cultures of DCs that had previously been stimulated with DNA vaccine or DNA vector, we observed that the CFU number was similar to that detected in unstimulated DC cultures. It is interesting to note that bacilli growth was higher in unstimulated macrophages than in unstimulated DCs, despite the fact that a similar number of bacilli were detected within both cells after 4 h of infection (12,1 × 10^4 ^± 5,9 × 10^4 ^and 10,7 × 10^4 ^± 6,1 × 10^4 ^CFU, respectively). These data show that while macrophages secreted high levels of TNF-alpha, IL-6 and IL-10 after DNA-HSP65 stimulation, DCs secreted IL-12 and up-regulated the expression of CD80, CD86 and CD83. Moreover, the stimulation of human macrophages with DNA-HSP65 seems to improve its microbicidal potential against *M. tuberculosis*, since we did not find significant difference between CFU numbers after 4 h and 7 d of infection. On the other hand, DCs were unable to kill intracellular *M. tuberculosis *after being stimulated with DNA-HSP65.

### Activation of the adaptive immune response induced by DNA-HSP65

**Figure 4:**
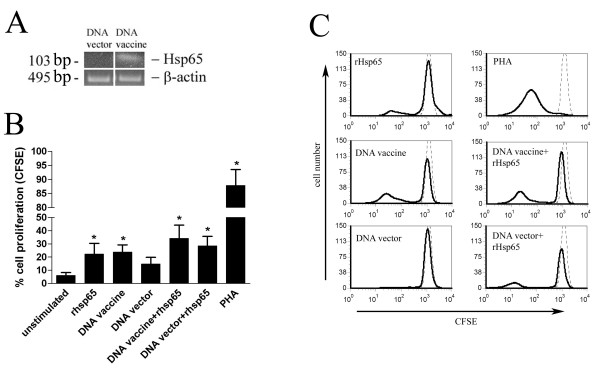
**Activation of adaptive immune response induced by DNA-HSP65**. (A) Expression of Hsp65 mRNA by monocytes stimulated with DNA vaccine or vector was evaluated by RT-PCR. (B) Proliferation of PBMCs after stimulation with DNA-HSP65. CFSE-labeled PBMCs were cultured with DNA vaccine, vector or with recombinant Hsp65 (rHsp65). Cell proliferation was determined by flow cytometry. Results represent the mean ± SEM of nine experiments. * p < 0,05, when compared to unstimulated cells. (C) Representative histograms of PBMCs proliferation assay. Black line: stimulated cells; dotted line: unstimulated cells.

To investigate the ability of DNA-HSP65 to activate adaptive immune response, the proliferation of PBMC induced by the DNA vaccine was determined. For this purpose, CFSE-labeled PBMC were stimulated with DNA vaccine or vector and analysed by flow cytometry. As positive control of specific proliferation, PBMC were stimulated with recombinant Hsp65 protein (rHsp65). The mRNA for Hsp65 was detected in monocytes cultured for 96 h with DNA-HSP65 (Figure 4A). DNA-HSP65 induced significant proliferation of PBMC compared to unstimulated cells. On the other hand, DNA vector was unable to induce a significant proliferation of PBMC (Figure 4B). Recombinant Hsp65 protein did not exhibit an additional effect on PBMC proliferation induced by DNA-HSP65. Figure 4C shows the histograms and is representative of one experiment. These data indicate that DNA-HSP65 is also able to activate the adaptive immune response leading to a Hsp65-specific cell proliferation.

## Discussion

In this study we not only verified that the DNA vaccine encoding the *Mycobacterium leprae *65 kDa heat shock protein (DNA-HSP65) was uptaken by human macrophages and DCs, but we also demonstrated that this vaccine induced a distinct pattern of cytokine production. Additionally, we showed that DNA-HSP65 induced an up-regulation of costimulatory molecules, changing the cell phenotype and improved the microbicidal activity of macrophages against *M. tuberculosis*. On top of that, DNA-HSP65 was able to induce specific cell proliferation.

The differential activation of macrophages and DCs described here may be related to their ability to uptake the vaccine. Despite the fact that almost 100% of macrophages were able to uptake DNA vaccine, our results showed that a subpopulation of DCs presented the highest ability to uptake the vaccine. Different endocytic mechanisms involving distinct receptors in each cell type may be related. Recently, it was described that a human keratinocyte cell-line is able to uptake plasmid DNA by a mechanism that involves macropinocytosis and binding to two DNA-binding cell surface proteins, ezrin and moesin [28]. In addition, some specific receptors, such as the macrophage class A scavenger receptor MARCO (macrophage receptor with a collagenous structure) are involved in the endocytosis of plasmid DNA by mouse peritoneal macrophages [29]. Since different receptors may be involved with the uptake of plasmid DNA, it is possible that distinct signalling pathways occur. Moreover, it was recently reported that the nature of pDCs response to TLR9 activation depends primarily on the intracellular compartment in which the CpG-TLR9 interaction occurs. The interaction of CpG-TLR9 at early endosomes induces IFN-alpha by pDCs, whereas CpG-TLR9 interaction at late endosomes promotes maturation of pDCs [30]. Thus, it is possible that monocyte-derived macrophages and DCs uptake DNA vaccine by different routes. Consequently, DNA may localize in distinct cellular compartments, generating different biological responses.

The type of DCs used in this study is also discussed. It was previously described that monocyte-derived DCs do not express TLR9 [31], so it was reasonable to assume that they were not activated by CpG-ODN. However, a recent report showed that monocyte-derived DCs contain TLR9 protein in amounts comparable with pDCs [32]. We have also observed that monocyte-derived DCs express intracellular TLR9 protein. These authors also described that monocyte-derived DCs captured CpG-ODN, secreted IFN-alpha and that CpG-ODN-stimulated DCs primed allogeneic CD4^+ ^T cells for proliferation and differentiation into IFN-gamma-secreting Th1 cells [32]. These data are in agreement with our results. However, we did not observe the IFN-alpha production by monocyte-derived DCs. In parallel with the cellular activation, we also verified that macrophages and DCs exhibited different microbicidal ability after being stimulated with DNA-HSP65. Despite the fact that macrophages stimulated with DNA-HSP65 were more effective to restrict the *M. tuberculosis *growth compared to DCs under the same stimulation, unstimulated macrophages presented higher mycobacterial growth than DCs. Two different groups have described that unstimulated DCs are more permissive to *M. tuberculosis *growth than macrophages [33,34]. However, an *in vivo *study that evaluated the DC functions after mycobacterial infection showed that BCG bacilli survive and remain stable in number inside DCs, suggesting that these cells may represent a hidden reservoir for mycobacteria [35]. Our data are concurring with these later authors.

Recent studies support the hypothesis that macrophages and DCs may have different roles during TB infection [36]. Therefore, the possibility of DNA-HSP65-stimulated macrophages and DCs present predetermined roles cannot be excluded. Giacomini *et al*. [36] described that after *M. tuberculosis *infection, the proinflammatory cytokines TNF-alpha, IL-1 and IL-6 and the immunosuppressive cytokine IL-10 were secreted mainly by monocyte-derived macrophages, while IL-12 was secreted almost exclusively by monocyte-derived DCs. They suggested that during *M. tuberculosis *infection macrophages secrete proinflammatory cytokines, whereas DCs are primarily involved in inducing antimycobacterial T cell immune response. Despite the fact that we studied the interaction of these APCs with a DNA vaccine, the same pattern of cellular activation reported by Giacomini *et al*. [36] was observed herein. On the other hand, other studies have shown that *M. tuberculosis *and *M. bovis *inhibit IL-12 secretion [37,38]. In this context, the observation that DNA-HSP65 stimulated IL-12 secretion by DCs is interesting and appears to support the hypothesis that this plasmid used as vaccine could be more useful to obtain a protective immune response than the infection itself.

It is important to mention that the stimulation induced by DNA vector was as effective as DNA vaccine regarding the cytokine production, expression of surface markers and microbicidal activity. This may be explained by the hypothesis that the immunostimulatory properties of either DNA vaccine or DNA vector described here are attributed to the presence of CpG ODN on plasmid backbone. A pattern consistent with CpG-driven immune activation was suggested by the comparable immune responses elicited by a vaccine encoding the circumsporozoite protein of *Plasmodium yoelii *and the plasmid backbone alone [39]. Our data are in agreement with these authors.

Finally, we demonstrated that DNA-HSP65 was able to induce significant proliferation of PBMC. Our results suggest that the cells that proliferated in response to DNA-HSP65 stimulation were Hsp65-specific, since both unstimulated and DNA vector-stimulated PBMC exhibited similar proliferation response. From the nine healthy individuals tested in these assays, six were tested for their reactivity against mycobacterial antigens (PPD test): three individuals were PPD^+ ^and three were PPD^-^. We found that both individuals – PPD^+ ^and PPD^- ^– displayed similar cell proliferation after stimulation with DNA-HSP65. In tuberculosis and leprosy patients, Hsp65-specific T cells have repeatedly been identified. Interestingly, T cells with reactivity to Hsp65 have also been identified in normal healthy individuals lacking any clinical signs of disease [40]. This demonstrates that Hsp65 is a prominent antigen that triggers a significant portion of the immune response, irrespective of whether the individual have already encountered or not this antigen.

## Conclusion

Overall, our results suggest that DNA-HSP65 is able to activate human immune response by different ways. Despite the fact that *in vitro *studies do not exactly mimic the microenvironmental conditions of *in vivo *studies, they do provide an approximation of how human APCs are activated *in vivo*. The data reported herein provide clues to the establishment of new strategies to improve APCs microbicidal activity. Finally, our findings have important implications for the design of new strategies based on immunotherapies and, consequently, on modulation of immune response in TB.

## Authors' contributions

Nine researchers participated in this study. LHF and VLDB are the principal investigators in this study. ELM provided the blood samples from Ribeirão Preto Haemocentre Foundation donors. CO and MCJ provided confocal and fluorescence microscopy analyses. PFW and APFT participated in the experiments of RT-PCR for mRNA Hsp65 detection. AAMC and CLS provided critical input and assistance. VLDB coordinated the project. All authors read and approved the final manuscript.

## References

[B1] Gurunathan S, Klinman DM, Seder RA (2000). DNA vaccines: immunology, application, and optimization*. Annu Rev Immunol.

[B2] Laddy DJ, Weiner DB (2006). From plasmids to protection: a review of DNA vaccines against infectious diseases. Int Rev Immunol.

[B3] Ishii KJ, Akira S (2006). Innate immune recognition of, and regulation by, DNA. Trends Immunol.

[B4] Zelenay S, Elias F, Flo J (2003). Immunostimulatory effects of plasmid DNA and synthetic oligodeoxynucleotides. Eur J Immunol.

[B5] Hacker H, Vabulas RM, Takeuchi O, Hoshino K, Akira S, Wagner H (2000). Immune cell activation by bacterial CpG-DNA through myeloid differentiation marker 88 and tumor necrosis factor receptor-associated factor (TRAF)6. J Exp Med.

[B6] Takeshita F, Ishii KJ, Ueda A, Ishigatsubo Y, Klinman DM (2000). Positive and negative regulatory elements contribute to CpG oligonucleotide-mediated regulation of human IL-6 gene expression. Eur J Immunol.

[B7] Takeshita F, Klinman DM (2000). CpG ODN-mediated regulation of IL-12 p40 transcription. Eur J Immunol.

[B8] Yamamoto M, Sato S, Mori K, Hoshino K, Takeuchi O, Takeda K, Akira S (2002). Cutting edge: a novel Toll/IL-1 receptor domain-containing adapter that preferentially activates the IFN-beta promoter in the Toll-like receptor signaling. J Immunol.

[B9] Wolff JA, Malone RW, Williams P, Chong W, Acsadi G, Jani A, Felgner PL (1990). Direct gene transfer into mouse muscle in vivo. Science.

[B10] Corr M, Lee DJ, Carson DA, Tighe H (1996). Gene vaccination with naked plasmid DNA: mechanism of CTL priming. J Exp Med.

[B11] Doe B, Selby M, Barnett S, Baenziger J, Walker CM (1996). Induction of cytotoxic T lymphocytes by intramuscular immunization with plasmid DNA is facilitated by bone marrow-derived cells. Proc Natl Acad Sci USA.

[B12] Iwasaki A, Torres CA, Ohashi PS, Robinson HL, Barber BH (1997). The dominant role of bone marrow-derived cells in CTL induction following plasmid DNA immunization at different sites. J Immunol.

[B13] Akbari O, Panjwani N, Garcia S, Tascon R, Lowrie D, Stockinger B (1999). DNA vaccination: transfection and activation of dendritic cells as key events for immunity. J Exp Med.

[B14] Klinman DM, Yi AK, Beaucage SL, Conover J, Krieg AM (1996). CpG motifs present in bacteria DNA rapidly induce lymphocytes to secrete interleukin 6, interleukin 12, and interferon gamma. Proc Natl Acad Sci USA.

[B15] Porgador A, Irvine KR, Iwasaki A, Barber BH, Restifo NP, Germain RN (1998). Predominant role for directly transfected dendritic cells in antigen presentation to CD8+ T cells after gene gun immunization. J Exp Med.

[B16] Ulmer JB, Deck RR, Dewitt CM, Donnhly JI, Liu MA (1996). Generation of MHC class I-restricted cytotoxic T lymphocytes by expression of a viral protein in muscle cells: antigen presentation by non-muscle cells. Immunology.

[B17] Bonato VL, Lima VM, Tascon RE, Lowrie DB, Silva CL (1998). Identification and characterization of protective T cells in hsp65 DNA-vaccinated and Mycobacterium tuberculosis-infected mice. Infect Immun.

[B18] Sedegah M, Hedstrom R, Hobart P, Hoffman SL (1994). Protection against malaria by immunization with plasmid DNA encoding circumsporozoite protein. Proc Natl Acad Sci USA.

[B19] Silva CL, Bonato VL, Lima VM, Faccioli LH, Leao SC (1999). Characterization of the memory/activated T cells that mediate the long-lived host response against tuberculosis after bacillus Calmette-Guerin or DNA vaccination. Immunology.

[B20] Ulmer JB, Donnelly JJ, Parker SE, Rhodes GH, Felgner PL, Dwarki VJ, Gromkowski SH, Deck RR, DeWitt CM, Friedman A, Hawe LA, Leander KR, Martinez D, Perry HC, Shiver JW, Montgomery DL, Liu MA (1993). Heterologous protection against influenza by injection of DNA encoding a viral protein. Science.

[B21] Bonato VL, Goncalves ED, Soares EG, Santos RR, Sartori A, Coelho-Castelo AA, Silva CL (2004). Immune regulatory effect of pHSP65 DNA therapy in pulmonary tuberculosis: activation of CD8+ cells, interferon-gamma recovery and reduction of lung injury. Immunology.

[B22] Lowrie DB, Tascon RE, Bonato VL, Lima VM, Faccioli LH, Stavropoulos E, Colston MJ, Hewinson RG, Moelling K, Silva CL (1999). Therapy of tuberculosis in mice by DNA vaccination. Nature.

[B23] Coelho-Castelo AA, Santos RR, Bonato VL, Jamur MC, Oliver C, Silva CL (2003). B-lymphocytes in bone marrow or lymph nodes can take up plasmid DNA after intramuscular delivery. Hum Gene Ther.

[B24] Calarota S, Bratt G, Nordlund S, Hinkula J, Leandersson AC, Sandstrom E, Wahren B (1998). Cellular cytotoxic response induced by DNA vaccination in HIV-1-infected patients. Lancet.

[B25] Hejdeman B, Bostrom AC, Matsuda R, Calarota S, Lenkei R, Fredriksson EL, Sandstrom E, Bratt G, Wahren B (2004). DNA immunization with HIV early genes in HIV type 1-infected patients on highly active antiretroviral therapy. AIDS Res Hum Retroviruses.

[B26] MacGregor RR, Boyer JD, Ugen KE, Lacy KE, Gluckman SJ, Bagarazzi ML, Chattergoon MA, Baine Y, Higgins TJ, Ciccarelli RB, Coney LR, Ginsberg RS, Weiner DB (1998). First human trial of a DNA-based vaccine for treatment of human immunodeficiency virus type 1 infection: safety and host response. J Infect Dis.

[B27] Wang R, Doolan DL, Le TP, Hedstrom RC, Coonan KM, Charoenvit Y, Jones TR, Hobart P, Margalith M, Ng J, Weiss WR, Sedegah M, de Taisne C, Norman JA, Hoffman SL (1998). Induction of antigen-specific cytotoxic T lymphocytes in humans by a malaria DNA vaccine. Science.

[B28] Basner-Tschakarjan E, Mirmohammadsadegh A, Baer A, Hengge UR (2004). Uptake and trafficking of DNA in keratinocytes: evidence for DNA-binding proteins. Gene Ther.

[B29] Jozefowski S, Sulahian TH, Arredouani M, Kobzik L (2006). Role of scavenger receptor MARCO in macrophage responses to CpG oligodeoxynucleotides. J Leukoc Biol.

[B30] Guiducci C, Ott G, Chan JH, Damon E, Calacsan C, Matray T, Lee KD, Coffman RL, Barrat FJ (2006). Properties regulating the nature of the plasmacytoid dendritic cell response to Toll-like receptor 9 activation. J Exp Med.

[B31] Jarrossay D, Napolitani G, Colonna M, Sallusto F, Lanzavecchia A (2001). Specialization and complementarity in microbial molecule recognition by human myeloid and plasmacytoid dendritic cells. Eur J Immunol.

[B32] Hoene V, Peiser M, Wanner R (2006). Human monocyte-derived dendritic cells express TLR9 and react directly to the CpG-A oligonucleotide D19. J Leukoc Biol.

[B33] Bodnar KA, Serbina NV, Flynn JL (2001). Fate of Mycobacterium tuberculosis within murine dendritic cells. Infect Immun.

[B34] Fortsch D, Rollinghoff M, Stenger S (2000). IL-10 converts human dendritic cells into macrophage-like cells with increased antibacterial activity against virulent Mycobacterium tuberculosis. J Immunol.

[B35] Jiao X, Lo-Man R, Guermonprez P, Fiette L, Deriaud E, Burgaud S, Gicquel B, Winter N, Leclerc C (2002). Dendritic cells are host cells for mycobacteria in vivo that trigger innate and acquired immunity. J Immunol.

[B36] Giacomini E, Iona E, Ferroni L, Miettinen M, Fattorini L, Orefici G, Julkunen I, Coccia EM (2001). Infection of human macrophages and dendritic cells with Mycobacterium tuberculosis induces a differential cytokine gene expression that modulates T cell response. J Immunol.

[B37] Gagliardi MC, Teloni R, Giannoni F, Pardini M, Sargentini V, Brunori L, Fattorini L, Nisini R (2005). Mycobacterium bovis Bacillus Calmette-Guerin infects DC-SIGN-dendritic cell and causes the inhibition of IL-12 and the enhancement of IL-10 production. J Leukoc Biol.

[B38] Mariotti S, Teloni R, Iona E, Fattorini L, Giannoni F, Romagnoli G, Orefici G, Nisini R (2002). Mycobacterium tuberculosis subverts the differentiation of human monocytes into dendritic cells. Eur J Immunol.

[B39] Mor G, Klinman DM, Shapiro S, Hagiwara E, Sedegah M, Norman JA, Hoffman SL, Steinberg AD (1995). Complexity of the cytokine and antibody response elicited by immunizing mice with Plasmodium yoelii circumsporozoite protein plasmid DNA. J Immunol.

[B40] Munk ME, Schoel B, Kaufmann SH (1988). T cell responses of normal individuals towards recombinant protein antigens of Mycobacterium tuberculosis. Eur J Immunol.

